# Identification of genetic and biochemical mechanisms associated with heat shock and heat stress adaptation in grain amaranths

**DOI:** 10.3389/fpls.2023.1101375

**Published:** 2023-02-02

**Authors:** Alejandra Reyes-Rosales, Gabriela Cabrales-Orona, Norma A. Martínez-Gallardo, Lino Sánchez-Segura, Jazmín P. Padilla-Escamilla, Paola A. Palmeros-Suárez, John P. Délano-Frier

**Affiliations:** ^1^ Departamento de Biotecnología y Bioquímica, Centro de Investigación y de Estudios Avanzados del Instituto Politécnico Nacional, Unidad Irapuato, Irapuato, Guanajuato, Mexico; ^2^ Departamento de Producción Agrícola, Centro Universitario de Ciencias Biológicas y Agropecuarias, Universidad de Guadalajara, Zapopan, Jalisco, Mexico

**Keywords:** grain amaranth, heat stress, heat shock, unknown-function genes, reproductive fitness

## Abstract

Heat stress is poised to become a major factor negatively affecting plant performance worldwide. In terms of world food security, increased ambient temperatures are poised to reduce yields in cereals and other economically important crops. Grain amaranths are known to be productive under poor and/or unfavorable growing conditions that significantly affect cereals and other crops. Several physiological and biochemical attributes have been recognized to contribute to this favorable property, including a high water-use efficiency and the activation of a carbon starvation response. This study reports the behavior of the three grain amaranth species to two different stress conditions: short-term exposure to heat shock (HS) conditions using young plants kept in a conditioned growth chamber or long-term cultivation under severe heat stress in greenhouse conditions. The latter involved exposing grain amaranth plants to daylight temperatures that hovered around 50°C, or above, for at least 4 h during the day and to higher than normal nocturnal temperatures for a complete growth cycle in the summer of 2022 in central Mexico. All grain amaranth species showed a high tolerance to HS, demonstrated by a high percentage of recovery after their return to optimal growing conditions. The tolerance observed coincided with increased expression levels of unknown function genes previously shown to be induced by other (a)biotic stress conditions. Included among them were genes coding for RNA-binding and RNA-editing proteins, respectively. HS tolerance was also in accordance with favorable changes in several biochemical parameters usually induced in plants in response to abiotic stresses. Conversely, exposure to a prolonged severe heat stress seriously affected the vegetative and reproductive development of all three grain amaranth species, which yielded little or no seed. The latter data suggested that the usually stress-tolerant grain amaranths are unable to overcome severe heat stress-related damage leading to reproductive failure.

## Introduction

The genus Amaranthus consists of *ca.* 60-70 of annual herbaceous plants with C4 photosynthesis (Kadereit et al., 2003; Hernández-Ledesma et al., 2015; Das, 2016). They are further divided into three sub-genera; *Amaranthus Albersia* (vegetable amaranth), *Amaranthus Amaranthus* (grain amaranth) and *Amaranthus Acnida* (weedy amaranth) (Sauer, 1950; Mosyakin and Robertson, 1996; Das, 2016). Their agricultural importance as vegetable and/or grain crops or herbicide-resistant weeds is widely recognized, although they may also be used as ornamentals or as a source of pigments (Shukla et al., 2003; Tranel and Trucco, 2009; Shukla et al., 2010; Achigan-Dako et al., 2014; Teng et al., 2015; Joshi et al., 2018; Hoidal et al., 2019; Sarker and Oba, 2019). The three grain amaranth species, i.e., *Amaranthus cruentus*, *A. hypochondriacus* and *A. caudatus*, are greatly valued for their highly nutritional, protein-rich seeds that also possess several nutraceutical properties (Písaříková et al., 2005; Caselato-Sousa and Amaya-Farfán, 2012; Rastogi and Shukla, 2013; Venskutonis and Kraujalis, 2013; Montoya-Rodríguez et al., 2015; De Ron et al., 2017). They are native of the American continent, although their origin and taxonomic relationships remain uncertain (2020; Trucco and Tranel, 2011; Kietlinski et al., 2014; Adhikary and Pratt, 2015; Stetter and Schmid, 2017; Stetter et al., 2017, 2020; Wu and Blair, 2017).

Grain amaranths are climate-resilient and stress-tolerant plants able to withstand drought, salinity, heat and ultraviolet irradiance. They also sustain severe defoliation, insect and/or pathogen damage and thrive in poor soils and low-input agricultural systems. This resilience has been associated with various anatomical, physiological and biochemical adaptations (2014; 2015; Miller et al., 1984; Lal and Edwards, 1996; Brenner et al., 2000; Johnson and Henderson, 2002; Délano-Frier et al., 2004; Liu and Stützel, 2004; Sánchez-Hernández et al., 2004; Omami and Hammes, 2006; Omamt et al., 2006; Aguilar-Hernández et al., 2011; Huerta-Ocampo et al., 2011; Caselato-Sousa and Amaya-Farfán, 2012; Castrillón-Arbeláez et al., 2012; Vargas-Ortiz et al., 2013; Vargas-Ortiz et al., 2015; Casarrubias-Castillo et al., 2014; González-Rodríguez et al., 2019; Jamalluddin et al., 2019; Cisneros-Hernández et al., 2021). Additional data from a large-scale transcriptomic analysis of *A. hypochondriacus* plants revealed the presence several orphan or unknown function genes that changed their expression levels in response to diverse stress conditions (Délano-Frier et al., 2011), a number of which were found to significantly increase (a)biotic stress tolerance when over-expressed in *Arabidopsis thaliana* (Massange-Sánchez et al., 2015; Massange-Sánchez et al., 2016; Palmeros-Suárez et al., 2015; Palmeros-Suárez et al., 2017; Cabrales-Orona, 2016; Cabrales-Orona, 2017; Cabrales-Orona, 2016; Cabrales-Orona, 2017; Cabrales-Orona and Délano-Frier, 2021; Cabrales-Orona et al., 2022).

Tolerance to heat stress is becoming an agronomic trait of critical importance given the worrying predictions of an increasingly endangered food security caused by damaging high-temperature events linked to the rise in the average global temperatures (Hoegh-Guldberg et al., 2018). These will inevitably affect worldwide crop and ecosystem productivity considering the deleterious impact that high-temperature stress has on practically all facets of plant development, growth and reproduction (Dusenge et al., 2019; Li et al., 2019; Ortiz-Bobea et al., 2019; Sun et al., 2022). Several studies have reported heat-stress resilience in diverse amaranth species, which has been associated with their ability to repair damaged tissues and to re-establish normal cellular and metabolic functions after heat stress exposure (Gou and Al-Khatib, 2003; Moran and Showeler, 2005). Regarding the latter, a recent study found that heat shock (HS) and a combination of drought and HS had differential, species-specific effects, on three grain amaranths and *A. spinosus*, a weedy amaranth (Netshimbupfe et al., 2022). Thus, the higher heat and drought tolerance shown by *A. cruentus* and *A. spinosus* compared to *A. caudatus* and *A. hypochondriacus* was attributed to a more stress-resilient photosynthetic apparatus, in addition to augmented proline (Pro) accumulation, higher relative water contents and reduced oxidative damage.

The present study further examined the response of grain amaranth to different heat-related stress conditions. Recovery rates and plant fitness after short- and long-term exposure to high temperatures were also recorded. The latter, in order to explore the potential climate resilience of grain amaranths, defined as their ability to overcome high temperature-related damage once the plants return to preferable conditions. Thus, *A. cruentus*, *A. caudatus* and *A. hypochondriacus* plants were subjected to both short-term HS and extended heat stress treatments (see Geange et al., 2021). All grain amaranth species showed high recovery rates after ca. 26 to 30 h periods of exposure to 45°C, although recovery was species-dependent, being significantly higher in *A. hypochondriacus.* Higher recovery from HS coincided with definite expression patterns of unknown function genes, previously shown to be induced by several other abiotic stresses, during both the HS treatment and the recovery period. HS recovery was also concurrent with the activation of certain antioxidant enzymes and the accumulation, Pro, phenols flavonoids, non-structural carbohydrates and pigments. Despite the fact the fatality rates were low, the performance of grain amaranths was seriously affected when kept under long-term heat stress. The overall negative effects observed were species specific, and included substantial modifications in vegetative growth and reproductive fitness. The evidence gathered indicates that grain amaranth may be able to withstand short periods of high temperatures without suffering major fitness penalties, but not sustained heat stress. It further supports the notion that grain amaranths are climate resilient crops that, similarly to other emerging crops like fonio (*Digitaria exilis*), taro (*Colocasia esculenta*), quinoa (*Chenopodium quinoa*) and a perennial wheatgrass (*Thinopyrum intermedium*), may readily adapt to the rapidly changing climatic conditions of the planet.

## Materials and methods

A summary of the experimental design of the present study, involving the use of several analytical strategies, is shown in **Figure S1** in **Supplementary Materials**

### Plant material

Three grain amaranth species, *Amaranthus hypochondriacus* cv. “Revancha”*, A. cruentus* cv. “Dorada” *and A. caudatus* were employed in this study. Plants were reproduced from seed stocks that originated from certified seeds provided by Dr. Eduardo Espitia Rangel, INIFAP, México, curator of the Mexican amaranth germplasm collection. Grain amaranth plants were used for experimentation at different points after germination (see below). The plants were germinated and grown in a conditioned growth chamber maintained under controlled conditions of light (photosynthetically active radiation ≈ 300 μmol m^−2^ s^−1^) photoperiod and temperature (16 h light/8 h dark at constant 28°C), as described previously (Palmeros-Suárez et al., 2015).

### Abiotic stress in grain amaranths: Salt and water deficit stress treatments

Five- to six-week-old *A. hypochondriacus* plants in the vegetative 2 (V2) stage, having 9-15 expanded leaves (Cisneros-Hernández et al., 2021) were used to perform water-deficit and salinity stress experiments, which were performed in the growth room conditions mentioned above. Ten to twelve plants per stress treatment were employed and each treatment was replicated twice. In brief, salt stress was imposed by watering the 1.3 L pots for eight consecutive days with 100 mL of a 400 mM NaCl solution. At this point, the electrical conductivity of the soil, measured at field capacity, was ≈ 6.8 dS/m. This is within the soil salinity range known to affect moderately salt-sensitive crops (http://www.fao.org/docrep/005/y4263e/y4263e0e.htm). Electrical conductivity was measured using a portable HI 98130 pH/conductivity/total dissolved solids water-proof tester (Hanna instruments Inc., Woonsocket, RI, USA). Control plants, maintained in the same conditions, were watered with deionized-distilled (dd) water only. The water deficit stress (WDS) treatments involved withholding irrigation for 8 days. The soil water potential gradually fell with increasing soil water loss as follows: -0.48 to -0.63 MPa, after 4 days; -0.96 to -1.057 MPa, after 6 days, and -1.45 to -1.68 MPa, after 8 days. The soil water potential was measured using an HR-33T dew point micro-voltmeter (Wescor Inc., Logan, UT, USA). Pools of combined leaf samples produced from two groups of three plants each were collected during both stress treatments at 4, 6 and 8 days and were immediately frozen in liquid N_2_ and subsequently stored at -80°C until required. Prior to analysis, the frozen samples were ground to a fine powder with mortar and pestle and under liquid N_2_.

### Abiotic stress in grain amaranths: Heat shock treatment

Heat shock (HS) is defined as the short-term exposure of a plant to severe high temperatures. The exposure lasts for minutes to a few hours and the air temperature increase is often 20°C or higher than the temperature range required for optimal plant development and reproduction (Mittler et al., 2012). Thus, HS experiments were performed with *A. hypochondriacus, A. cruentus and A. caudatus* plants at the V1 development stage (Cisneros-Hernández et al., 2021) which defines grain amaranth plants having 6 to 8 expanded leaves. All HS experiments were performed in a growth chamber kept at 45°C and under constant illumination. The exposure time to 45°C was established independently for each grain amaranth species. It was determined by the hours needed to produce evident heat stress-related wilting in at least half of the population sample, which consisted of 30 plants. Thus, the HS treatments applied, at 45°C, were as follows: 26 h for *A. cruentus* and 30 h for *A. hypochondriacus* and *A. caudatus*. All plants were subsequently allowed to recover for 4 days under the optimal growth conditions mentioned above, time after which the percentage of plants that recovered was recorded. Additional time-course HS experiments were performed in which pooled leaf samples from 2 groups of 3 grain amaranth plants each were collected after different exposure periods to 45°C: 1, 3, 6, 12, 24 and 26 h, for *A. cruentus*, and 1, 3, 6, 12, 24 and 30 h, for *A. hypochondriacus* and *A. caudatus*. Leaf samples of identical number of plants from all three grain amaranth species were also collected 1 and 3 and days after recovery.

### Abiotic stress in grain amaranths: Long-term heat stress

Chronic heat stress experiments were performed with 12 plants of each of the three grain amaranth species examined. Plants were germinated in a germination tray as mentioned above. They were transplanted to 1.5 L pots filled with a general soil mix (Palmeros-Suárez et al., 2015) when the seedlings reached the V1 development stage, 32 days after germination. The plants were immediately transferred to the greenhouse where the heat stress experiment was conducted (see below) and maintained as such for 14 days. They were then transferred to 14 L plastic bags to finalize the stress treatment. The stress treatment had a duration of 63 days; it was started in August 1, 2022 and concluded in October 3, 2022. Seed harvest was done sometime later, between October 11 and November 15, 2022. The temperature and relative humidity (RH) inside the greenhouse were recorded daily in the early morning (7 a.m.), in mid-afternoon (3 p.m.) and in the evening (6:30 p.m.). The average temperatures and RHs for the duration of the stress treatment were the following: 23.9°C and 75%, at 7 a.m.; 52.6°C and 21%, at 3 p.m., and 38.5°C and 35%, at 6:30 p.m. The average temperatures and RHs under which non-stressed control plants were maintained for the same time span were the following: 21.7°C and 91%, at 7 a.m.; 35.2°C and 65%, at 3 p.m., and 32.6°C and 59%, at 6:30 p.m. Plant survival was recorded at the end of the experiment. Plant height was recorded thrice, 10, 22 and 57 days after the start of the stress treatment and leaf number was determined only once, after 57 days of stress. The days to panicle emergence and subsequent panicle growth, flowering and seed formation were also monitored. Photographic record of the control and stressed plants was performed twice, on September 22, 2022 and September 30, 2022. All plants were watered to field capacity for the duration of the experiment.

### Quantitative PCR assays

Total RNA was extracted and purified from 100-200 mg of frozen and ground grain amaranth foliar tissues as described by Palmeros-Suárez et al. (2015). The integrity of the purified RNA was assessed by denaturing electrophoresis in ethidium bromide-containing formaldehyde/formamide 1% agarose gels, while RNA purity and concentration were determined in a NanoDrop 2000 apparatus (ThermoFisher Scientific; Waltham, MA, USA). cDNA was synthesized from 4 μg of total RNA using 200 units of the SuperScript II reverse transcriptase as instructed by the manufacturer (Invitrogen, Carlsbad, CA, USA). The cDNA produced was diluted 25-fold with dd water prior to quantitative PCR (qPCR) analyses, which were performed using SYBR Green detection chemistry and a CFX96 Real Time System (Bio-Rad, Hercules, CA, USA) and the primers listed in **Table S1** in **Supplementary Materials**. These were designed using the following programs: *Primer3* (http://bioinfo.ut.ee/primer3/), *Beacon Designer* (http://www.premierbiosoft.com), UNAFold (http://www.idtdna.com/UNAFold) and Oligo Evaluator (http://www.oligoevaluator.com) (Thornton and Basu, 2011). Reaction mixtures, 20 μL total volume, contained 2 μL cDNA solution, 4 μL of each 2 μM oligonucleotide solutions, 8 μL de Sybr Green Jumpstart TAQ Ready Mix (Sigma-Aldrich St. Louis, MO, USA) and 2 μL of sterile dd water. The amplification process was performed as follows: 15 min at 95°C to activate the Taq Polymerase, followed by 40 cycles of denaturation (95°C/15 s) annealing (60°C/1 min) and extension (72°C/30 s) and a final extension step (60°C/1 min). The expression levels of several unknown function grain amaranth genes were determined in leaves of grain amaranth plants subjected to drought, salt and/or HS stress.

The criteria used to select the genes tested were the following: 1) Previous experimental evidence of their induced expression in response to several (a)biotic stress conditions in grain amaranth (Délano-Frier et al., 2011) and other plants, and 2) presumable participation in the regulation and/or responses designed to protect plants from heat-related stress conditions. The analysis included *AhHAB4-PAI-1* (Phytozome 13, v2.1, accession number: AH018279) coding for an RNA- and hyaluronan-binding protein; *AhRIP* (Phytozome 13, v2.1, accession number: AH007263) coding for an RNA-editing factor interacting protein involved in chloroplast and mitochondrial RNA editing, similar to a protein recently found to participate in mitochondrial development in salt-stressed *A. thaliana* (Jiang et al., 2021); *Ah2880* (Phytozome 13, v2.1, accession number: AH015341) coding for a small unknown function protein that, similarly to *AhRIP*, was shown to be differentially induced in grain amaranths by various biotic and abiotic stress conditions (Délano-Frier et al., 2011; Cabrales-Orona and Délano-Frier, 2021); *AhBAMY* (Phytozome 13, v2.1, accession number: AH001114), coding for a β-amylase that was shown to provide heat stress-resistance when over-expressed in *A. thaliana* (Cabrales-Orona, 2016); *AhDGR2* (Phytozome 13, v2.1, accession number: AH015369) coding for a DUF642 protein that altered root growth and cell wall structure when overexpressed in transgenic *A. thaliana* plants (Palmeros-Suárez et al., 2017); *AhTIL* (Phytozome 13, v2.1, accession number: AH014131) coding for a temperature-induced lipocalin, similar to proteins known to provide thermo-tolerance in *A. thaliana* (Chi et al., 2009); *AhERD* (Phytozome 13, v2.1, accession number: AH006452), coding for a senescence/dehydration-associated protein similar to EARLY RESPONSIVE TO DEHYDRATION 7, ERD7, one of several proteins identified in lipid droplets, i,e. neutral-lipid-containing organelles isolated from drought-stressed *A. thaliana* leaves (Doner et al., 2021), and *AhOEE* (Phytozome 13, v2.1, accession number: AH015200), coding for an oxygen evolving enhancer protein found to be strongly affected by (a)biotic stresses in *A. hypochondriacus* (Délano-Frier et al., 2011). This protein is similar to a photosystem II component that regulates the function of photosystem II, *via* the optimization of the manganese cluster during water photolysis, in *A. thaliana* and tobacco (Heide et al., 2004). It is also presumed to protect the D1 reaction center against oxidative damage during drought and heat stress in different algal and plant models (Yamamoto, 2001; Kim et al., 2015; Cevik et al., 2019; Li et al., 2021a). The expression levels of these genes were normalized in relation to their expression in leaves of control plants, which was set to a value of 1.0. Relative gene expression was calculated using the 2^-ΔΔC^_T_ comparative cycle threshold method (Livak and Schmittgen, 2001). The genes employed as normalizing controls were the *AhACT7* actin and *Ahβ-tubulin* housekeeping genes. In all cases, leaves for qPCR assays were obtained from the grain amaranth plant pools generated during the stress treatments, as described above. Each pool was subsequently subjected to four independent sampling procedures prior to analysis. qPCR data are reported as the mean of 4 technical replicates ± SE of each experiment. qRT-PCR expression analyses were validated in two independent experiments.

### Measurement of biochemical parameters

Several biochemical parameters were analyzed in grain amaranth plants subjected to HS and recovery treatments. The methods used were described previously by Cabrales-Orona et al. (2022) and Portillo-Nava et al. (2021). These included the following: total betalains, carotenes and chlorophyll; soluble and insoluble non-structural carbohydrates (NSCs) and Pro levels and total phenols and flavonoids.

The same extracts used for the analysis of total phenol and flavonoid leaf contents (Sakanaka et al., 2005) were used for the subsequent HPLC analysis of phenylpropanoids and phenolic acids (method 1, M1) and flavonoids (method 2, M2), respectively. These analyses were performed only with leaf extracts obtained from *A. hypochondriacus* plants maintained in optimal conditions, subjected to HS for 30 h or after a 3-day recovery period following HS. M1 was performed on a 250 × 4.6 mm I.D. (particle size, 5µm; pore size, 120Å) YMC-Pack ODS-AM reversed-phase C_18_ column (YMC CO., LTD., Kyoto, Japan) equipped with a C_18_ guard cartridge, using an Agilent 1200 HPLC system (Agilent Technologies; Santa Clara, CA, USA). The temperature of the column oven was kept at 25°C. A gradient elution was employed with a mobile phase consisting of 1% glacial acetic acid (solution A) and HPLC-grade acetonitrile (solution B) as follows: linear gradient to 5% B, 0-5 min; linear gradient from 5% B to 10% B, 5-10 min; linear gradient from 10% B to 15% B, 10-30 min; isocratic elution at 15% B, 30-40 min; linear gradient from 15% B to 50% B, 40-60 min, and linear gradient from 50% B to 100% B, 60-90 min. A 2 min post-time linear gradient from 100% B to 0% B was included before the next injection. The mobile phase flow rate and the sample injection volume were 1.0 mL/min and 10 μL, respectively. All quantifications were based on peak areas. Gallic acid (retention time, [Rt], 9.36 min), hydroxybenzoic acid (Rt, 18.65 min), chlorogenic acid (Rt, 19.97 min), catechin (Rt, 19.77 min), vanillic acid (Rt, 22.07 min), caffeic acid (Rt, 23.68 min), epicathequin (Rt, 26.74 min), vanillin (Rt, 29.04 min), coumaric acid (Rt, 33.15 min), rutin (Rt, 83.89 min), myricetin (Rt, 83.90 min) and kaempferol (Rt, 83.92) were used as standards.

M2 was performed on a 150 × 4.6 mm I.D. (particle size, 5 µm; pore size, 120Å) Zorbax Eclipse XDB reversed-phase C_18_ column (Agilent Technologies) equipped with a C_18_ guard cartridge, using an Agilent 1200 HPLC system (Agilent Technologies). The temperature of the column oven was kept at 25°C. A gradient elution was employed with a mobile phase consisting of water: acetic acid: acetonitrile (88:2:10 *v*/*v*/*v*; solution A) and water: acetic acid: HPLC-grade acetonitrile (8:2:90 *v*/*v*/*v*; solution B) as follows: linear gradient to 15% B, 0-5 min; linear gradient from 15% B to 50% B, 5-20 min; linear gradient from 50% B to 70% B, 20-25 min; linear gradient from 70% B to 100% B, 25-30 min; isocratic elution at 100% B, 30-40 min. A 5 min post-time linear gradient from 100% B to 0% B was included before the next injection. The mobile phase flow rate and the sample injection volume were 1.0 mL/min and 20 μL, respectively. All quantifications were based on peak areas. Chlorogenic acid (Rt, 3.95 min), catechin (Rt, 5.15 min), rutin (Rt, 6.99 min), myricetin (Rt, 9.78 min) and kaempferol (Rt, 15.39) were used as standards.

### Antioxidant enzymes and H_2_O_2_

Catalase (CAT), superoxide dismutase (SOD) and glutathione reductase (GR) enzyme activities, as well as hydrogen peroxide levels, were also determined. The enzymology methods employed were previously described by Cabrales-Orona et al. (2022) and Palmeros-Suárez et al. (2015). H_2_O_2_ was determined by the colorimetric quantification of a xylenol orange and ferric iron complex produced after the peroxide-dependent oxidation of ferrous iron (National Diagnostics, Atlanta, GA, USA).

### Statistical analysis

All the experiments were performed using a completely randomized design with at least 6 plants per genotype. Data generated were analyzed using a one-way analysis of variance (ANOVA) to examine: 1) the effect of HS stress on the expression levels of the 8 unknown function genes in grain amaranth plants; 1) the significance of the extent of recovery observed after HS, and 3) the effect on various biochemical variables recorded in grain amaranth plants subjected to HS. The means were compared using the Tukey-Kramer test to identify statistically significant differences between them. The statistical analysis was performed with the aid of R (http://r-project.org/) Rstudio (https://www.rstudio.com) and JMT Pro13 (jmp.com) statistical software.

## Results

### Responsiveness of selected grain amaranth unknown function genes to high salt and water-deficit stresses

**Figure 1:**
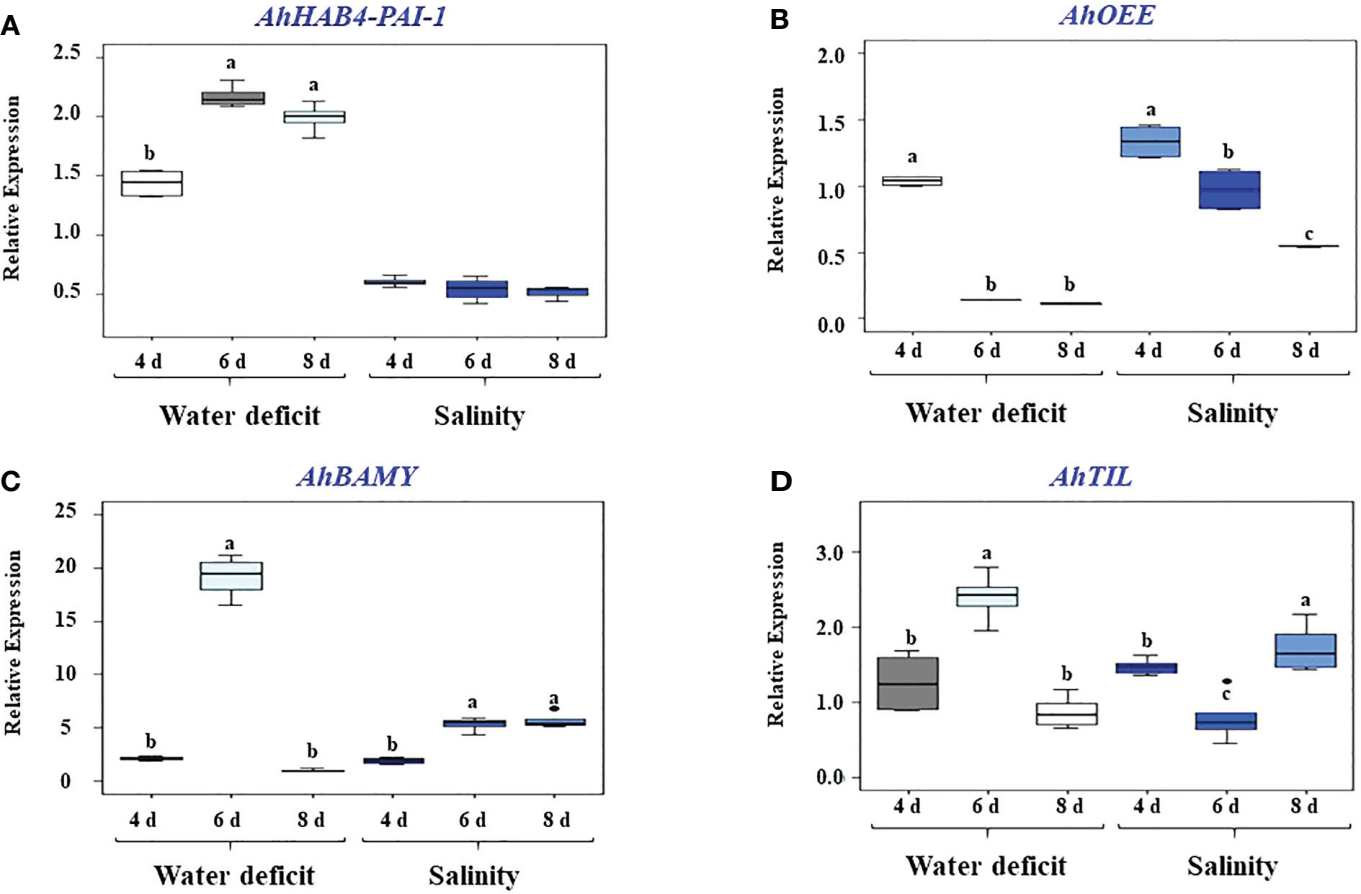
Effect of high salt and water-deficit stress on the expression levels of four grain amaranth unknown function genes. Relative gene expression was quantified in leaves of grain amaranth plants subjected to high salt or water deficit stress for 4, 6 and 8 days, respectively. The three recognized grain amaranth species were analyzed: *Amaranthus hypochondriacus* cv. Revancha*, A cruentus* cv. Dorada *and A caudatus*. Foliar RNA was employed for the qRT-PCR analysis of presumably stress-inducible unknown function genes, namely: **(A)** *AhHAB4-PAI-1*; **(B)** *AhOEE;* **(C)** *AhBAMY*, and **(D)** *AhTIL*. Threshold cycles (C_T_) values for all genes were normalized to the C_T_ value of the *AhACT7* and *AhEF1a* housekeeping genes. Changes in gene expression were calculated from 4 technical replicates of pools prepared from 10-to-12 plants sampled per time point using the 2^-ΔΔCT^ method (Livak and Schmittgen, 2001). Different letters over the box-and-whisker plots, showing high, low, and median values, represent statistically significant differences; one-way ANOVA and Tukey Kramer test*, P* < 0.05.

Further analysis of four stress-responsive unknown function genes was performed in this work. The selected genes (i.e., *AhHAB4-PAI-1*, *AhBAMY, AhTIL* and *AhOEE*) were subjected to excess salt and WDS treatments to corroborate the only reported evidence of their responsiveness to these compromising conditions (Délano-Frier et al., 2011). Thus, the results from the quantitative gene expression assays shown in **Figure 1C** show that *AhBAMY* was strongly induced by salt stress, reaching *ca.* 6-fold higher expression levels at the end of the treatment. Conversely *AhTIL* (**Figure 1D**) was weakly induced after 8 days of salt stress, whereas *AhOEE*, and particularly *AhHAB4-PAI-1*, were repressed (**Figures 1A, B**). *AhOEE* was negatively affected by WDS, becoming repressed by the sixth day of WDS exposure, whereas *AhHAB4-PAI-1* responded positively to WDS, reaching more than 2-fold higher expression levels at the same time point (**Figures 1A, B**). *AhTIL* and *AhBAMY* were more responsive to WDS than to salt stress, with both genes reaching highest expression levels 4 days after water was withheld from the plants. *AhBAMY*, in particular, showed a heightened response to these two stress conditions, particular WDS (**Figures 1C, D**).

### Responsiveness of selected grain amaranth unknown function genes to heat shock and subsequent recovery

The foliar expression of all the unknown function grain amaranth genes tested in plants exposed to HS and subsequent recovery was distinctly different (**Table 1**). HS tolerance in the three grain amaranth species was associated with an early induction of the RNA binding (*AhHAB4-PAI-1*) and RNA editing (*AhRIP*) genes. These were found to peak between 3 and 6 h after the start of the HS treatment followed by a gradual decrease of their expression levels that extended to the end of the HS treatments (i.e., 26 to 30 h). These diminished gene expression levels were, nevertheless, still significantly higher than those recorded in control plants. A stronger induction of these two genes, and of *AhHAB4-PAI-1* in particular, was also detected during the recovery period, especially 1 day after returning to optimal conditions. The *AhERD* gene was also found to be extensively induced in response to the HS treatment in all grain amaranths. However, contrary to the latter two genes, the expression levels were much more intensely induced during the early stages of the HS treatment, except in *A. caudatus*, rather than during recovery. *AhTIL* and *AhDGR* produced expression patterns that were similar to *AhERD*, being expressed almost exclusively during HS conditions, mostly at early time points, and remaining unchanged, and even repressed in *A. cruentus* and *A. hypochondriacus*, during the late HS stages and/or the recovery periods. The expression pattern of these genes in *A. caudatus* differed again from those recorded in the other two species. *Ah2880* was exclusively induced during the HS stress, but contrary to the above three genes, its induction was detected at the late stages of the HS treatment. *AhBAMY* was transiently expressed within the first 12 h of the HS treatment and was later extensively repressed, except for the brief induction recorded 1 day after recovery in *A. hypochondriacus*. Finally, a strong repression of the *AhOEE* gene was first observed 6 h after the onset of the HS treatment in *A. cruentus* and *A. caudatus*; this effect extended for the rest of the HS treatment and included the recovery period. In contrast, *AhOEE* was induced within the first 12 h of HS stress in *A. hypochondriacus* and subsequently returned to levels that were no different from those recorded in control plants, except for a short-lived repression observed 24 h after HS treatment.

**Table 1:** Effect of heat shock and subsequent recovery on the expression of grain amaranth unknown function genes.

	Heat shock						Recovery	
*A. hypochondriacus*								
**GENE/time**	**1 h**	**3 h**	**6 h**	**12 h**	**24 h**	**30 h**	**1 day**	**3 days**
*AhHAB4-PAI-1*	**4.65 ± 0.29** ^cde^	**15.20 ± 2.68** ^b^	**11.88 ± 2.02** ^bc^	**5.12 ± 0.60** ^cde^	**8.17 ± 0.98b** ^cd^	*0.05 ± 0.01* ^e^	**47.62 ± 2.81** ^a^	**4.15 ± 0.56b** ^de^
*AhRIP*	**1.68 ± 0.23** ^cd^	**3.25 ± 0.41** ^b^	**2.48 ± 0.15** ^bc^	1.41 ± 0.09^de^	1.12 ± 0.05^de^	0.79 ± 0.05^de^	**4.14 ± 0.17** ^a^	0.73 ± 0.01^e^
*AhERD*	**13.34 ± 1.14** ^a^	**5.07 ± 0.56** ^b^	**1.71 ± 0.21** ^c^	1.41 ± 0.114^c^	**11.37 ± 0.12** ^a^	0.96 ± 0.124^c^	**1.90 ± 0.28** ^c^	0.83 ± 0.08^c^
*AhTIL*	**3.08 ± 0.44** ^a^	1.36 ± 0.22^bc^	1.03 ± 0.3^bc^	**1.77 ± 0.20** ^b^	0.91 ± 0.07^bc^	1.08 ± 0.07^bc^	0.62 ± 0.06^c^	0.99 ± 0.04^bc^
*AhDGR*	**3.24 ± 0.53** ^b^	**5.38 ± 0.29** ^a^	**2.50 ± 0.24** ^b^	0.96 ± 0.07^c^	0.73 ± 0.13^c^	0.89 ± 0.13^c^	1.29 ± 0.15^c^	*0.55 ± 0.02* ^c^
*AhBAMY*	*0.55 ± 0.05* ^c^	1.34 ± 0.10^b^	1.04 ± 0.10^b^	1.08 ± 0.09^b^	*0.16 ± 0.03* ^c^	*0.24 ± 0.03* ^c^	**1.85 ± 0.07** ^a^	*0.23 ± 0.01* ^c^
*Ah2880*	1.14 ± 0.09^d^	1.43 ± 0.08^cd^	**1.74 ± 0.12** ^bc^	**1.59 ± 0.03** ^cde^	**2.25 ± 0.10** ^ab^	**2.57 ± 0.16** ^a^	1.35 ± 0.07^cd^	*0.51 ± 0.06* ^e^
*AhOEE*	**3.92 ± 0.21** ^a^	**4.41 ± 0.19** ^a^	**2.20 ± 0.06** ^b^	**1.88 ± 0.24** ^bc^	*0.02 ± 0.04* ^e^	0.70 ± 0.04^d^	1.46 ± 0.13^c^	0.79 ± 0.08^d^
*A. cruentus*								
**GENE/time**	**1 h**	**3 h**	**6 h**	**12 h**	**24 h**	**26 h**	**1 day**	**3 days**
*AhHAB4-PAI-1*	**2.61 ± 0.34** ^cd^	**5.23 ± 0.82** ^c^	**15.16 ± 1.88** ^b^	1.10 ± 0.21^cd^	0.63 ± 0.08^d^	**2.47 ± 0.12** ^cd^	**20.21 ± 0.49** ^a^	**14.38 ± 1.10** ^b^
*AhRIP*	**1.59 ± 0.08** ^bcd^	**2.46 ± 0.09** ^b^	**5.00 ± 0.20** ^a^	**1.98 ± 0.81** ^bc^	0.74 ± 0.20^cd^	1.33 ± 0.20^bcd^	**1.85 ± 0.44** ^bcd^	*0.26 ± 0.02* ^d^
*AhERD*	**40.86 ± 0.96** ^a^	**26.12 ± 1.81** ^a^	**7.50 ± 0.89** ^d^	**18.04 ± 0.85** ^c^	**5.52 ± 0.79** ^de^	**14.44 ± 0.79** ^c^	**2.85 ± 0.21** ^e^	**1.56 ± 0.14^e^**
*AhTIL*	**4.76 ± 0.57** ^a^	**3.34 ± 0.39** ^b^	**2.11 ± 0.18** ^b^	*0.53 ± 0.13* ^c^	0.61 ± 0.04^c^	*0.52 ± 0.04* ^c^	*0.28 ± 0.05* ^c^	*0.28 ± 0.06* ^c^
*AhDGR*	**1.83 ± 0.08** ^b^	**3.71 ± 0.89** ^a^	**3.40 ± 0.10** ^a^	*0.48 ± 0.14* ^bc^	*0.12 ± 0.02* ^c^	*0.05 ± 0.02* ^c^	*0.35 ± 0.06* ^bc^	*0.11 ± 0.01* ^c^
*AhBAMY*	0.78 ± 0.051^bc^	**2.41 ± 0.22** ^a^	**2.43 ± 0.20** ^a^	0.82 ± 0.09^b^	*0.52 ± 0.13* ^bc^	*0.54 ± 0.13* ^bc^	0.97 ± 0.28^b^	*0.05 ± 0.01* ^c^
*Ah2880*	*0.50 ± 0.05* ^d^	1.15 ± 0.04^c^	1.39 ± 0.08^bc^	**1.89 ± 0.15** ^ab^	0.90 ± 0.07^cd^	**2.33 ± 0.24** ^a^	0.86 ± 0.07^cd^	*0.46 ± 0.05* ^d^
*AhOEE*	**1.73 ± 0.05** ^a^	0.64 ± 0.05^b^	*0.41 ± 0.05* ^c^	*0.11 ± 0.01* ^d^	*0.01 ± 0.00* ^d^	*0.008 ± 0.00* ^d^	*0.08 ± 0.01* ^d^	*0.15 ± 0.02* ^d^
*A. caudatus*								
**GENE/time**	**1 h**	**3 h**	**6 h**	**12 h**	**24 h**	**30 h**	**1 day**	**3 days**
*AhHAB4-PAI-1*	**5.08 ± 0.60** ^c^	**20.30 ± 0.89** ^ab^	**24.09 ± 2.61** ^a^	1.19 ± 0.23^c^	0.76 ± 0.10^c^	*0.06 ± 0.010* ^c^	**26.36 ± 1.63** ^a^	**14.38 ± 2.21** ^b^
*AhRIP*	0.74 ± 0.02^d^	**1.59 ± 0.12** ^cd^	**4.91 ± 0.38** ^a^	**3.94 ± 0.22** ^ab^	**2.35 ± 0.25** ^bc^	**1.80 ± 0.25** ^cd^	**3.15 ± 0.51** ^bc^	**3.02 ± 0.38** ^bc^
*AhERD*	**9.64 ± 0.70** ^bc^	**6.26 ± 1.17** ^bcd^	**12.08 ± 0.32** ^b^	**26.24 ± 0.87** ^a^	**26.47 ± 1.47** ^a^	**28.38 ± 1.47** ^a^	**4.70 ± 0.62** ^cd^	**1.83 ± 0.07** ^d^
*AhTIL*	**4.26 ± 0.38** ^bc^	1.31 ± 0.15^ef^	**5.08 ± 0.24** ^ab^	**6.11 ± 0.47** ^a^	**3.19 ± 0.41** ^cd^	**4.04 ± 0.41^b^** ^c^	**2.63 ± 0.24** ^de^	0.99 ± 0.15^f^
*AhDGR*	0.68 ± 0.14^c^	1.25 ± 0.08^b^	**1.88 ± 0.09** ^a^	1.35 ± 0.07^ab^	0.90 ± 0.01^bc^	*0.54 ± 0.01* ^c^	0.99 ± 0.07^bc^	0.66 ± 0.03^c^
*AhBAMY*	*0.23 ± 0.00* ^de^	*0.03 ± 0.01* ^ef^	**2.17 ± 0.15** ^a^	**1.65 ± 0.11** ^b^	*0.27 ± 0.00* ^cde^	*0.14 ± 0.00* ^e^	*0.55 ± 0.02* ^c^	*0.57 ± 0.04* ^cd^
*Ah2880*	*0.53 ± 0.05* ^c^	**1.70 ± 0.14** ^a^	*0.54 ± 0.07* ^c^	**1.72 ± 0.10** ^a^	**2.36 ± 0.23** ^a^	**2.21 ± 0.13** ^a^	1.42 ± 0.17^ab^	*0.45 ± 0.01* ^bc^
*AhOEE*	1.00 ± 0.02^a^	0.68 ± 0.09^b^	*0.02 ± 0.00* ^c^	*0.05 ± 0.00* ^c^	*0.03 ± 0.00* ^c^	*0.03 ± 0.00* ^c^	*0.01 ± 0.00* ^c^	*0.02 ± 0.00* ^c^

^a^Relative expression was quantified based on the 2^-ΔΔCT^ calculation (Livak and Schmittgen, 2001)

^b^Numbers in bold and in italics represent upregulated and down-regulated gene expression, respectively

Relative gene expression^a^ in leaves was quantified during heat shock treatment and subsequent recovery. Three grain amaranths species were employed: *Amaranthus hypochondriacus, Amaranthus cruentus and A. caudatus*.

**Figure 2:**
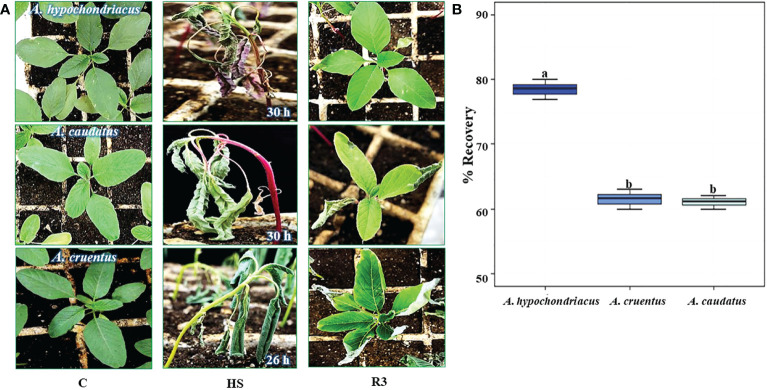
Heat shock tolerance of young grain amaranth plants. **(A)** Young grain amaranth plants, at the vegetative 1 stage of development, were placed in growth chamber and subjected to different heat shock (HS) periods, at 45°C and constant light: 30 h (*Amaranthus hypochondriacus* and (*A caudatus*) and 26 h (*A. cruentus*). The aspect of the stressed plants (HS, middle panel) was compared with untreated control, **(C)**, plants maintained in optimal growing conditions (left-hand panel) and with plants that recovered from HS, shown after a 3-day period in optimal conditions (R3, right-hand panel). **(B)** The box-and-whisker plots show the percentage of recovery recorded in each of the three grain amaranth species analyzed. Different letters over the box-and-whisker plots, showing high, low, and median values, represent statistically significant differences; one-way ANOVA and Tukey Kramer test*, P* < 0.05 (n = 30).

The highest recovery to HS, detected in *A. hypochondriacus* (**Figures 2A, B**), coincided with defined differences in the magnitude and timing of two of the genes analyzed: 1) a longer induction period of *AhHAB4-PAI-1* during HS, which was sustained for 24 h, in addition to very high expression levels during the early recovery period, which were *ca.* 2-fold higher than those recorded in the other two grain amaranth species, and 2) a prolonged induction of the *AhOEE* gene, which remained induced for 12 h under HS, compared to *A. cruentus* and *A. caudatus*, where the expression of this gene began to be strongly repressed by the sixth hour of HS stress.

### Biochemical changes produced in heat-shocked grain amaranth plants: Non-structural carbohydrates and Pro

**Figure 3:**
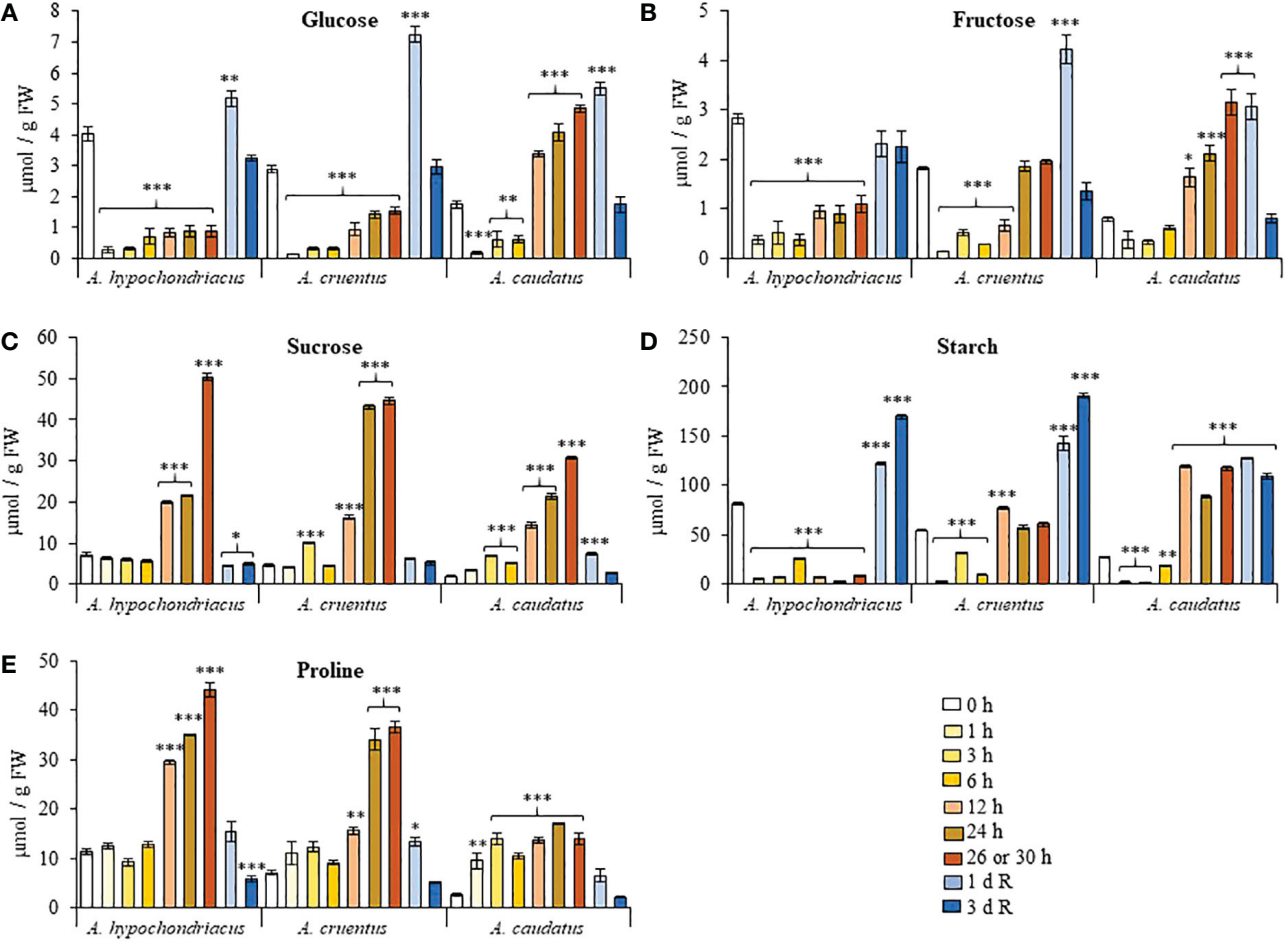
Fluctuation in the levels of non-structural carbohydrates and proline recorded during heat shock and subsequent recovery in grain amaranth plants. Changes in the levels of **(A)** glucose, **(B)** fructose, **(C)** sucrose, **(D)** starch and **(E)** proline detected in leaves sampled from grain amaranth plants at the V1 development stage subjected to heat shock (HS) for 0, 1,3, 6, 12, 24, 26 (*Amaranthus cruentus*) or 30 h (*A. hypochondriacus* and *A caudatus*) and after 1 (1 d R) and 3 (3 d R) days of recovery from HS under optimal conditions. The bars represent the mean value (n = 30) ± SE. Asterisks over the bars represent statistically significant differences within species at *P* ≤ 0.05 (*), *P* ≤ 0.01 (**) or *P* ≤ 0.001 (***); one-way ANOVA and Tukey Kramer test.

Most biochemical responses activated in response to heat stress shared a certain degree of similarity in *A. hypochondriacus* and *A. cruentus* and tended to differ, in some cases notably, in *A. caudatus*. Glucose (Glc) levels were reduced ca. 2 to 10-fold in heat-shocked *A. hypochondriacus* and *A. cruentus* plants (**Figure 3A**). The decrease in fructose (Fru) and starch contents followed a similar pattern, except *in A. cruentus* plants, where these NSCs increased to levels similar to, or higher (i.e., starch, at 12 h), than those detected in untreated plants during the final stages of the HS treatment (24 to 26 h) (**Figures 3B, D**). In contrast, a consistent increase of Glc, Fru and starch, that was first detected 12 h after the start of the HS treatment and persisted until its termination, was observed in *A. caudatus*. No species-specific differences were observed for sucrose (Suc), whose HS-induced levels started to increase significantly after 12 h and gradually reached maximum levels at the end of the HS treatment (**Figure 3C**). Non-species-specific effects also included the reduction of NSC contents that was recorded during the recovery period, as follows: i) a peak of Glc and Fru levels occurring 1 day after recovery followed to a return to control levels after day 3; ii) maximum starch accumulation, 1 day (in *A. caudatus*) or 3 days (in *A. hypochondriacus* and *A. cruentus*) after recovery (**Figures 3A, B, D**), and iii) the return of Suc to similar, or slightly higher levels, than those detected in control untreated plants (**Figure 3C**). Parallel to Suc, Pro accumulated at the final stages of the HS treatment and returned to levels similar to those in untreated control plants during the recovery period (**Figure 3E**).

### Total pigments: Carotenes, chlorophyll and betalains

**Figure 4:**
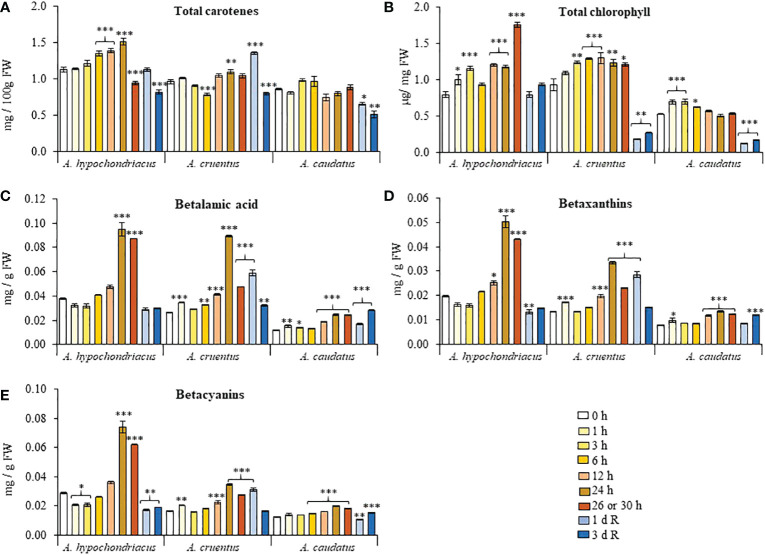
Fluctuation in the levels of different pigments recorded during heat shock and subsequent recovery in grain amaranth plants. Changes in the levels of **(A)** total carotenes, **(B)** total chlorophyll, **(C)** betalamic acid, **(D)** betaxanthins and **(E)** betacyanins detected in leaves sampled from grain amaranth plants at the V1 development stage subjected to heat shock (HS) for 0, 1,3, 6, 12, 24, 26 (*Amaranthus cruentus*) or 30 h (*A. hypochondriacus* and *A caudatus*) and after 1 (1 d R) and 3 (3 d R) days of recovery from HS under optimal conditions. The bars represent the mean value (n = 30) ± SE. Asterisks over the bars represent statistically significant differences within species at *P* ≤ 0.05 (*), *P* ≤ 0.01 (**) or *P* ≤ 0.001 (***); one-way ANOVA and Tukey Kramer test.

HS-induced changes in total carotene content differed between species (**Figure 4A**). In *A. hypochondriacus*, carotene content reached significantly higher levels than untreated controls 6 to 24 h after HS, only to decline at the end of the HS treatment and during recovery. In *A. cruentus*, total carotene content remained mostly unchanged during HS and peaked briefly, 1 day after recovery, whereas carotene content was similarly unaffected by HS in *A. caudatus* but was reduced during recovery. In all grain amaranths, carotene content was significantly lower than untreated controls 3 days after recovery. Total chlorophyll steadily increased concomitantly with HS, for the duration of the treatment in *A. hypochondriacus* and *A. cruentus*, and only for the first 6 h of HS exposure in *A. caudatus* (**Figures 4B**). Chlorophyll contents were reduced to similar or lower levels than those detected in untreated controls during the recovery period. Betalain pigments, and their betalamic acid precursor, showed a similar accumulation pattern, gradually increasing concomitantly with the duration of the HS treatment followed by a drastic reduction occurring 1 and/or 3 days after recovery (**Figures 4C–E**). HS-induced betalain pigment accumulation was ostensibly weaker in *A. caudatus*.

### Total phenols and flavonoids, antioxidant enzyme activity and H_2_O_2_ accumulation

**Figure 5:**
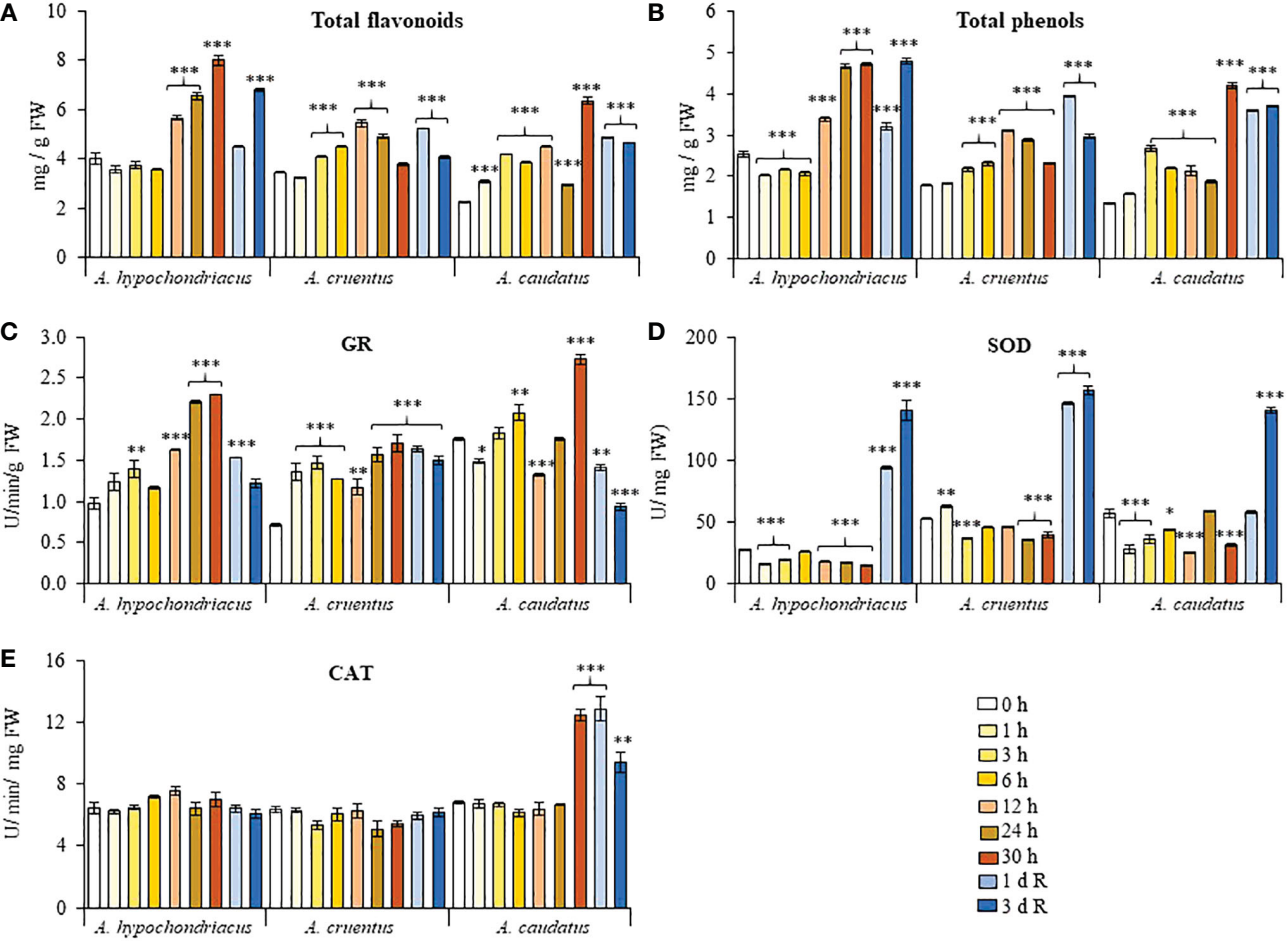
Fluctuation in the levels of antioxidant compounds and enzymes recorded during heat shock and subsequent recovery in grain amaranth plants. Changes in the levels of **(A)** total flavonoids, **(B)** total phenols, **(C)** glutathione reductase, GR, activity **(D)** superoxide dismutase, SOD, activity and **(E)** catalase, CAT, detected in leaves sampled from grain amaranth plants at the V1 development stage subjected to heat shock (HS) for 0, 1,3, 6, 12, 24, 26 (*Amaranthus cruentus*) or 30 h (*A. hypochondriacus* and *A caudatus*) and after 1 (1 d R) and 3 (3 d R) days of recovery from HS under optimal conditions. The bars represent the mean value (n = 30) ± SE. Asterisks over the bars represent statistically significant differences within species at *P* ≤ 0.05 (*), *P* ≤ 0.01 (**) or *P* ≤ 0.001 (***); one-way ANOVA and Tukey Kramer test.

Total phenols and flavonoids gradually increased in response to HS in all grain amaranths tested and peaked at the latter stages of their respective treatments. Flavonoid and phenol levels did not increase further during recovery, but remained significantly higher than those detected in untreated control plants, except in *A. hypochondriacus* and *A. caudatus* sampled 1 and 3 days after recovery, respectively (**Figures 5A, B**). This tendency was replicated in the HPLC analysis of *A. hypochondriacus* leaf extracts obtained from control and heat-shocked plants, respectively, sampled at the end of the HS treatments and after a 3-day recovery period. Thereby, the results shown in **Tables S2**, **S3** indicate that the leaves of heat-shocked plants produced the largest number of peaks, the majority of which had higher peak areas than those detected in control and recovered plant leaf samples. Only a handful of peaks were more abundant, or exclusive of, control and/or recovered plants. Curiously, none of the most abundant peaks produced in response to HS could be identified by comparison with the retention times of standard compounds. Also, the presence of late-eluting peaks using the M2 separation was indicative of possible modifications of phenols and/or flavonoids by methylation, acylation, glycosylation, conjugation, oxidation and/or other chemical reactions. The identification of these compounds is now in progress.

Antioxidant enzyme activities responded differently, and in a species-dependent manner, to the HS treatment and subsequent recovery. SOD activity was mostly reduced or unaffected during HS but underwent an increase in activity during the recovery period in all grain amaranths (**Figure 5C**). GR, in contrast, reached highest levels of activity at the end of the HS treatment and subsequently receded during recovery, except in *A. cruentus*, where the increase in GR activity during HS did not follow a uniform pattern and where no decrease in GR activity was detected during recovery (**Figure 5D**). CAT activity was predominantly unresponsive to HS and also remained unchanged during recovery, except in *A. caudatus*, where significant peaks in activity were observed 30 h after HS and during the following recovery period (**Figure 5E**). No H_2_O_2_ was detected in any of the grain amaranth leaf samples analyzed.

### Morphological and physiological alterations produced in chronically heat-stressed grain amaranth plants

**Figure 6:**
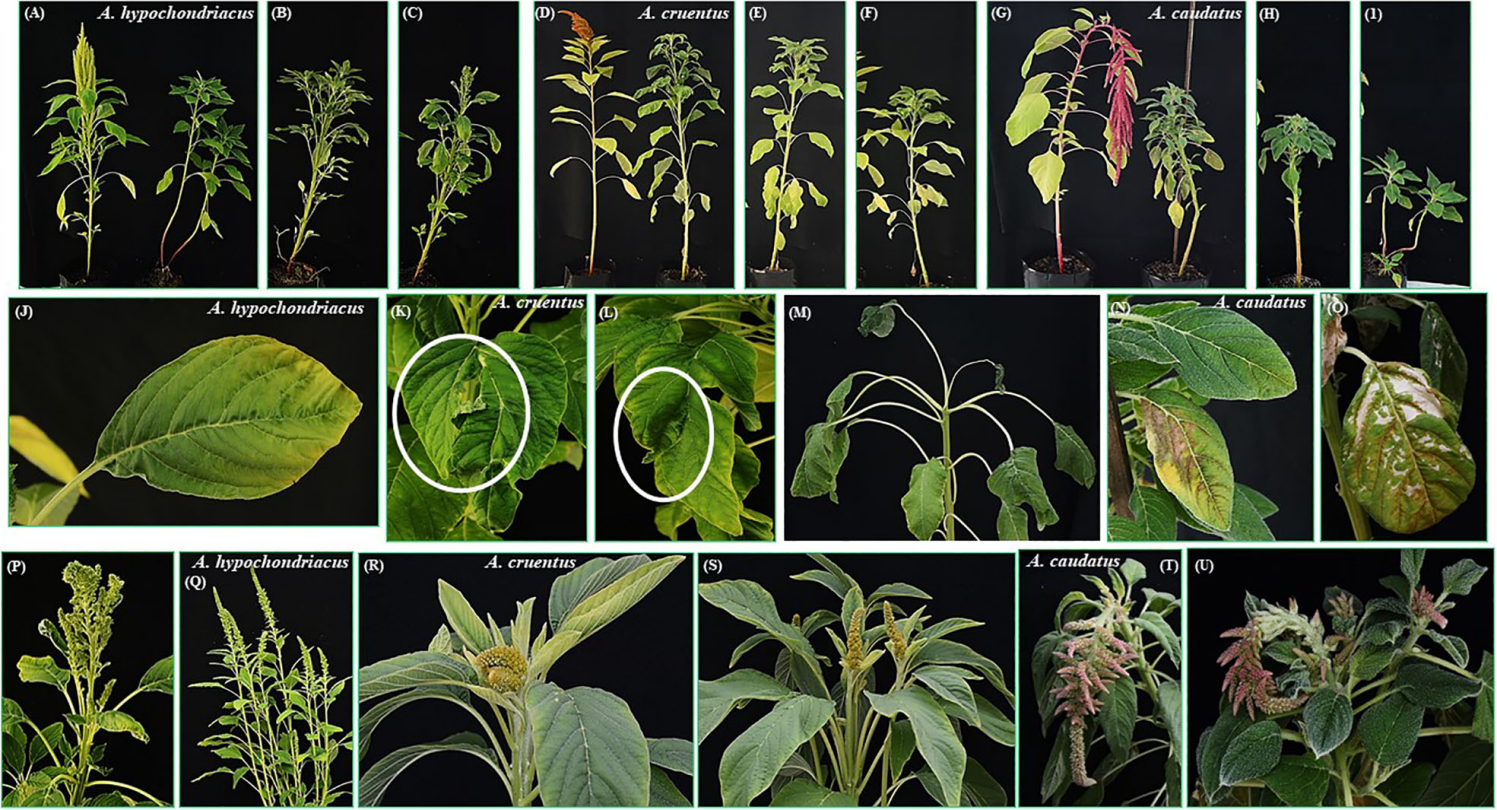
Effects of prolonged, severe heat stress on vegetative and reproductive development in grain amaranth plants. Severe long-term heat stress stunted plant growth, leading to plants that were, in average 1.5 to 2-fold shorter than control plants kept under optimal conditions **(A–I)**. Some heat-stressed plants ramified into two-stemmed plants, as shown in *A. hypochondriacus* **(A)** and *A. caudatus* **(I)**; Heat stress conditions caused leaf damage in *A. caudatus* **(J, N, O)**, or led to a morphological alteration characterized by the division of the leaf into two parts along the middle of the leaf surface **(K, L)**. Panicle emergence was greatly delayed or suppressed (i.e., in *A. cruentus*) **(M)**, and subsequent development was very slow (i.e., in *A. cruentus*) **(R)** or produced deformed panicles (i.e., in *A. hypochondriacus*) **(P)**. Flowering in the panicles produced by *A. cruentus* and *A. caudatus* was also delayed **(R, U)**, whereas flowering panicles (i.e., in *A. hypochondriacus* **(Q)** and *A. cruentus*) produced very low numbers of immature/mature seeds, or no seeds at all (*A. caudatus*). Heat stress also led to profuse branching, a modification that occurred concomitantly with panicle emergence in most plants of the three grain amaranth 3 species **(Q–U)**.

Although the survival to prolonged and severe heat stress in the three grain amaranth species tested was elevated (i.e., between 85 and 100%), the plants nevertheless showed severe responses to this condition that were manifested during both vegetative and reproductive development stages (**Table 2**; **Figure 6**). Heat-stressed plants were, in average, 1.5 to 2-fold shorter than control plants kept under optimal conditions (**Table 2**; **Figures 6A–I**). Plant height hindrance started early, being already evident 10 days after the exposure to stress (**Table 2**). Some heat-stressed *A. hypochondriacus* and *A. caudatus* plants ramified into two or three-stemmed plants (**Figures 6A–I**); the additional stems were always generated from early, and usually irreversible, heat stress-derived damage to the apical meristem of the main stem.

**Table 2:** Effect of extended, severe heat stress on vegetative and reproductive development parameters in grain amaranth species.

	*A. hypochondriacus*	*A. cruentus*	*A. caudatus*
Plant survival (%)^a^	88	100	82
Plant height (cm), 10 days^b^ (control, C)	34.33	32.50	19.1
Plant height, 10 days (heat stress, HS)	18.21	19.38	13.6
Plant height, 22 days^c^ (C)	61.61	66.70	57.0
Plant height, 22 days (HS)	41.43	56.38	31.0
Plant height, 57 days^c^ (C)	144.13	149.85	135.4
Plant height, 57 days (HS)	100.43	115.43	90.4
Leaf number (C)^d^	35.86	20.40	35.6
Leaf number (HS)	53.00	32.43	40.6
Panicle emergence (C)^e^	42	56	52
Panicle emergence (HS)	67	86	77
Panicle size, cm (C)^f^	40.94	26.80	41.3
Panicle size, cm (HS)	10.86	1.14	6.8
Flowering (C)^g^	8	14	16
Flowering, (HS)	10	22	20
Immature seed formation, (C)^h^	20	27	35
Immature seed formation (HS)	25	30	ND
Seed yield per plant, in g (C)^i^	11.98	7.6	6.13
Seed yield per plant, in g (HS)	3.43	1.07	ND

a Plant survival: determined 67 days after the start of the heat stress treatment.

b Average plant height: recorded 10 days after the start of the heat stress treatment, in plants grown in 1.5 L pots.

c Plant height, recorded after different heat-stress periods, in plants grown in 14 L plastic bags.

d Leaf number recorded after 57 days of the heat stress treatment, in plants grown in 14 L plastic bags.

e Panicle emergence was registered in days after germination.

f Panicle size was recorded 57 days after the start of the heat stress treatment.

g Flowering was recorded in days after panicle emergence (dape).

h Seed formation/maturation was recorded in dape.

i Seed yield represents the average seed yield per plant calculated from groups of 10-11 control plants and 7 surviving heat-stressed plants.

ND = Not detected.

In contrast, all heat-stressed plants produced a greater number of leaves, a parameter that was determined 57 days after the beginning of the heat stress treatment (**Table 2**). However, the leaves showed evident signs of damage, particularly in *A. caudatus* (**Figures 6J, N, O**), whereas some *A. cruentus*´ leaves developed a morphological alteration characterized by the division of the leaf into two sections, which emerged from a profound partition, resembling a mechanical rupture, that emerged along the middle of the leaf surface (**Figures 6K, L**). The reproductive performance of heat-stressed grain amaranths was also profoundly perturbed. Thus, panicle emergence was greatly delayed, or sometimes completely suppressed (i.e., in *A. cruentus*, **Figure 6M**), and, later, the emerging panicles had, in average, an extremely slow growth rate (i.e., in *A. cruentus*; **Figure 6R**) and some were deformed (i.e., in *A. hypochondriacus*; **Figure 6P**). Moreover, flowering in the panicles produced by *A. cruentus* and *A. caudatus* was delayed (**Figures 6R–U**), whereas flowering panicles in *A. hypochondriacus* were slow to produce mature seeds, as determined 60 days after heat stress (**Figure 6Q**; **Table 2**). At this stage, all control plants had produced mature seeds. Another striking effect associated with the reproductive performance of grain amaranths was the profuse branching that occurred concomitantly with panicle emergence in most plants of all 3 species (**Figures 6Q, S, U**). Seed yields in *A. hypochondriacus* and *A. cruentus* were 3.5 to 7-fold lower, respectively, than those produced by equivalent control plants, whereas no seeds were produced by heat-stressed *A. caudatus* plants (**Table 2**).

## Discussion

Grain amaranths have been demonstrated to be climate-resilient crops capable of adapting to harsh ambient conditions that are usually unfavorable to several commercial crops, including most cereals, on which the world´s food security depends. Various previous studies using grain amaranths as model plants have demonstrated that abiotic stress tolerance is highly species specific. This trait appears to occur as a consequence of the differential manifestation of various biochemical, molecular and physiological responses designed to reduce, for example, the negative impact of severe defoliation (2015; Vargas-Ortiz et al., 2013; Cisneros-Hernández et al., 2021), WDS (González-Rodríguez et al., 2019) and long-term flooding and hypoxia/anoxia (J.P. Padilla-Escamilla, personal communication), among other damaging ambient conditions. This study extended this perspective by concentrating on the effects and species-specific responses of grain amaranths to HS and heat stress conditions. A number of previous reports have recognized the capacity of amaranth plants, mostly vegetable and weed types, to withstand high-temperature stress, usually together with water-deficit conditions. For instance, studies performed with various *Amaranthus* species, revealed a differential capacity to repair damaged tissues after heat stress exposure and to subsequently reactivate normal metabolic functions, such as photosynthesis. Thus, part of the greater growth of *A. palmeri* at higher temperatures, compared to *A. retroflexus* and *A. rudis* was attributed to its extensive root growth and to the greater thermostability of its photosynthetic apparatus (Gou and Al-Khatib, 2003). Further species-specific differences in heat tolerance were recently associated with a higher tolerance of the photosynthetic apparatus of *A. cruentus* and *A. spinosus* to combined heat and drought stress, compared to *A. caudatus* and *A. hypochondriacus*. The difference observed was linked to a higher PSII photochemical efficiency and to an increased pool size of the final electron acceptors of PSI. Additional contributing factors were the accumulation of higher Pro levels and a higher membrane stability, which was correlated to a significantly reduced propensity to electrolyte leakage (Netshimbupfe et al., 2022). Another investigation found that seeds of *A. tricolor* and *A. spinosus* were able to germinate in conditions of extreme ground temperatures and low soil water potentials. This ability was interpreted as an adaptative trait to the high-temperature conditions of their natural tropical habitats, similar to several other intrusive plant species that take advantage of this capacity to enhance their invasive capacity (Ye and Wen, 2017). In this respect, a recent study suggested a link between the extent of the globulin seed protein fraction and the suitability of seeds of diverse grain amaranth species for use in arid regions of Mexico and other world regions (Barba de la Rosa et al., 2009). This proposal was based on the high homology of the globulin seed protein fraction with the Cupin domain of seed storage proteins of other plant species, considering that the latter has been associated with resistance to extreme heat and WDS (Khuri et al., 2001).

In the present study, a significantly higher tolerance to HS was observed in *A. hypochondriacus*. This characteristic was in accordance with the superior tolerance of this grain amaranth species to WDS compared to *A. cruentus* and *A. caudatus* (González-Rodríguez et al., 2019). Several biochemical responses, mostly detected in roots, were associated with the latter trait, including a stronger expression of abscisic acid marker genes, a more robust sugar starvation response and an enhanced osmotic adjustment. The latter was proposed to result from higher basal and WDS-induced hexose levels and hexose/sucrose ratios that coincided with a depletion of starch reserves in leaves and roots and augmented levels of raffinose family oligosaccharides and Pro. Although most of the biochemical and enzymatic parameters analyzed in this study were found to change drastically during HS and subsequent recovery, it was nevertheless found that, similar to WDS, greater HS tolerance in *A. hypochondriacus* compared to the other two grain amaranth species coincided with a number of specific parameters such as: i) a very pronounced depletion of leaf starch reserves during the HS treatment, and ii) a significantly higher accumulation of foliar Pro, Suc, total phenols and flavonoids and betalains and other pigments at the latter stages of the HS treatment. This pattern coincided with the greater accumulation of redox-sensitive phenols and flavonoids recently reported in WDS-tolerant accessions of *A. hypochondriacus* (Aditya et al., 2022). Likewise, a comparison of thermosensitive and thermotolerant lettuce cultivars revealed that the latter differentially accumulated specific phenylpropanoid and flavonoid compounds known to have strong antioxidant activity, upon heat treatment (Oh et al., 2009; Wei et al., 2021). Augmented Pro levels were also congruent with this compound’s role in the mitigation of high temperature stress and subsequent recovery due to its quenching of singlet oxygen and superoxide radicals (Kavi Kishor et al., 2022).

Modified levels of soluble sugars are similarly known to be altered during heat stress in order to regulate osmotic pressure within the cell (Wang et al., 2020). Moreover, soluble carbohydrate accumulation, mostly as Suc, is related to heat stress-activated starch degradation (Thalmann et al., 2016) coupled to enhanced sucrose phosphate synthase activity (Der Agopian et al., 2011). Starch breakdown has also been linked to the activation of β-amylase gene expression (see below) and activity, which is known to be induced by several stress conditions, including heat (Dreier et al., 1995). β-amylase activity also leads to the accumulation of maltose, which acts as a precursor of soluble sugar metabolism and as a protective agent of proteins, membranes and the photosynthetic electron transport chain under either heat or freezing stress (Kaplan and Guy, 2004; Kaplan et al., 2006). In general, the fluctuation of NSCs levels in leaves of heat-shocked grain amaranths was in agreement with the significant effect that high-temperature stress is known to have on photosynthesis, starch synthesis, Suc synthesis and transport, and photo-assimilate accumulation (Lal et al., 2022).

Regarding pigment levels, prior studies have revealed that wheat cultivars resistant to heat stress maintained higher total chlorophyll levels in addition to other favorable responses such as higher Fv/Fm ratios and increased photosynthetic and transpiration rates and stomatal conductance (Sharma et al., 2015). These findings were in accordance with previously reported data linking delayed senescence and heat tolerance in wheat (Reynolds et al., 1997; Vijayalakshmi et al., 2010). Further, transgenic sweet-potato plants overexpressing a modified *Orange* gene encoding a plastid-localized DnaJ protein known to regulate carotenoid synthesis and abiotic stress resistance, accumulated significantly higher total carotenoid and β-carotene contents in storage roots and leaves. Transgenic plants also showed greater tolerance to heat stress compared to untransformed WT plants. This finding agreed with the role of carotenoids in protecting the photosynthetic apparatus from photo-oxidative stress (Stanley and Yuan, 2019; Kim et al., 2021). Finally, stress-induced accumulation of betalain pigments, usually found in red-leafed betalain-accumulating plants adapted to harsh ambient conditions, was congruent with their protective effect against oxidative damage, a property linked to their to their superior ability to scavenge oxygen free radicals and to regulate cell osmotic pressure, similarly to Pro (Sdouga et al., 2019).

This study also expanded the information regarding the stress-responsive nature of various grain amaranth unknown function genes reported in prior studies (Délano-Frier et al., 2011; Cabrales-Orona and Délano-Frier, 2021). Thus, WDS was found to induce the expression of *AhHAB4-PAI-1*, *AhBAMY* and *AhTIL*, which reached their highest levels at an intermediate stage of the WDS treatment applied, whereas these genes, except *AhBAMY*, were mostly unsensitive to high salt stress. Conversely, *AhOEE* was strongly repressed by WDS and was also quite insensitive to salt stress. These results coincided with the perceived role of these genes in the regulation of drought tolerance in other plant models (Todaka et al., 2000; Levesque-Tremblay et al., 2009; Abo-Ogiala et al., 2014; Ambrosone et al., 2015; Zanella et al., 2016) and with the high stress sensitivity and concomitant inhibition of the oxygen-evolving complex (Hajheidari et al., 2005; Zadražnik et al., 2019).

The expression pattern of the genes analyzed in this work was also greatly affected during the HS treatment and the subsequent recovery period. The expression of several of them was congruent with strong experimental evidence describing their role in the protection of plants against abiotic stress, including heat-related damage, such as *AhERD*, commonly used as a marker for stress responses in other plant models (Cheng et al., 2013; Doner et al., 2021; Huang et al., 2021) and *AhTIL* (Levesque-Tremblay et al., 2009; Boca et al., 2014; Wahyudi et al., 2020). The heat responsiveness of the *Ah2880* gene was unexpected. However, this gene codes for a small protein that, similarly to lipocalins, could function by binding to other proteins and/or hydrophobic molecules during stress-amelioration cellular processes (Sanchez et al., 2006). This possibility remains to be tested experimentally, although its overexpression in transgenic *A. thaliana* plants led to significantly increased recovery to HS conditions, similar to the overexpression of *AhHAB4-PAI-1* coding for RNA-binding protein (Cabrales-Orona et al., in preparation; see below). Other genes examined showed a species-specific expression pattern that could offer clues as to the mechanisms leading to the higher HS tolerance observed in *A. hypochondriacus* plants. Therefore, the expression *AhHAB4-PAI-1* in leaves of heat-shocked *A. hypochondriacus* leaves was stronger and more persistent compared to *A. cruentus* and *A. caudatus*. Furthermore, the prominent expression peak of this gene, detected during the first stages of recovery in the three grain amaranths tested, was ca. 2-fold higher in *A. hypochondriacus*. The high *AhHAB4-PAI-1* expression detected in response to HS was congruent with recent data showing that the D2 and D4 RNA-binding and glycine-rich proteins from *A. thaliana*, coded by the *RBGD2* and *RBGD4* genes that share similarity with *AhHAB4-PAI-1*, are essential for heat tolerance in this plant model *via* their role in the organization of specific proteins and transcripts into stress granules. These are membrane-less condensates that assemble as a result of protein liquid-liquid phase separation in response to stress and are composed of un-translated mRNAs, translation initiation factors, proteins with intrinsically disordered regions and RNA-binding domains, in addition to several other components. Stress granules have been demonstrated to be crucial for adaptative cellular responses to stress and for post-stress recovery, when the release to the cytoplasm of mRNA and proteins from disassembling stress granules reactivates translation to restore cell growth and development (Maruri-López et al., 2021; Zhu et al., 2022). This proposal is supported by the highly increased recovery after heat-shock observed in transgenic *A. thaliana* plants overexpressing *AhHAB4-PAI-1* (Cabrales-Orona et al., in preparation). Other differences in the expression pattern of grain amaranth unknown function genes that coincided with the increased HS-tolerance detected in *A. hypochondriacus* were the slightly higher expression levels of the *AhRIP* and *AhDGR* genes during the first 3 h of the HS treatment, the more persistent induction of the *AhOEE* during the HS period and the induction of *AhBAMY* observed 1 day after recovery, but not during the HS treatment. These results are supported by data suggesting that RNA editing rates may affect heat stress responses in plants, such as *A. thaliana* and grape (Chu et al., 2020; Zhang et al., 2020). RNA editing proteins have also been recognized as part of the stress granules assembled in the cytoplasm and in the chloroplasts in response to HS conditions (Chodasiewicz et al., 2020). The proposed contribution of the *AhDGR* gene to increased HS tolerance in grain amaranths coincides with experimental evidence showing that cell wall remodeling through the regulation of its methylesterification levels influences plant responses to heat stress, e.g., by regulating the flexibility of the guard cell walls that is needed for their appropriate response to high temperature conditions (Wu et al., 2017, 2018; 2022;). Induced expression of *AhOEE* in leaves of *A. hypochondriacus*, after HS exposures that repressed this gene in the other two grain amaranth species, may have reflected an increased ability of its photosynthetic machinery to withstand anomalously high temperatures, considering that PSII, including the oxygen evolving complex, is considered to be one of the primary targets of high temperature stress, together with ATP generation and carbon fixation (Allakhverdiev et al., 2008; Barta et al., 2010). Thus, increased tolerance to HS in grain amaranths could be related to a higher photosynthetic activity under heat stress conditions, similarly to what was recently reported in maize and transgenic glycine-betaine-accumulating transgenic tomato plants (Doğru, 2021; Li et al., 2021b). Apart from the functional properties mentioned above, differential *AhBAMY* expression patterns could have favored the superior recovery of heat-shocked *A. hypochondriacus* plants by increasing the photochemical efficiency of the PSII in the chloroplast, similar to certain plastidic *BAMY* genes that have been found to enhance freezing stress in *A. thaliana* plants *via* this mechanism (Monroe et al., 2014).

Finally, the prolonged exposure to intense heat stress conditions had a negative effect on the vegetative and reproductive development of grain amaranth plants (Hedhly et al., 2009; Zhao et al., 2020). The impact observed was, once again, species dependent. Thus, *A. caudatus* showed a stronger susceptibility to heat stress, a trait possibly associated with its origin in the higher-altitude regions of Bolivia, Peru, and Ecuador, and/or to the fact that, in contrast to *A. hypochondriacus* and *A. cruentus*, it appears to have weaker domestication traits (Stetter et al., 2017). The strong influence that heat stress had on the reproductive phase of grain amaranths was in accordance to observations made in most other crop plants, in which a drastic reduction of seed yield was produced once their range of tolerable temperatures was exceeded (Porter, 2005). This is a sobering finding that should be taken into account when considering the use of grain amaranths as crops fit to sustain the increasingly warm conditions caused by the ongoing global climate change.

## Conclusions

The present study presents further experimental evidence supporting the resistance to heat-stress related damage usually attributed to grain amaranths. Several biochemical and molecular factors were found to be possible contributors to heat stress tolerance in these plants. Nevertheless, the species-specific rates of recovery observed after aggressive HS conditions that are lethal to several other model plants were in accordance with previously reported differences in grain amaranth tolerance to WDS and defoliation, among others. They further support the notion that grain amaranths have gradually acquired differential strategies to cope with (a)biotic stress despite their common ancestry, as suggested, for instance, by the association found between the differential expression levels of genes coding for RNA binding and RNA editing proteins, believed to play a crucial role in the plant’s responses to heat stress and to augment HS tolerance. Conversely, grain amaranths were strongly affected by chronic and severe heat stress conditions that are predicted to prevail in certain regions of the planet if the increase in global temperatures is not controlled. This is a cause for concern, considering that grain and vegetable amaranths have been lately proposed as heat-resilient candidate crops that may be able to thrive in the adverse climatic scenarios predicted for the near future.

## Data availability statement

The original contributions presented in the study are included in the article/**Supplementary Material**. Further inquiries can be directed to the corresponding authors.

## Author contributions

Conceived and designed the experiments: JD-F, PP-S, GC-O, AR-R, JP-E, and LS-S. Performed the experiments: GC-O, AR-R, JP-E, LS-S, and NM-G. Analyzed the data: JD-F, PP-S, NM-G, GC-O, AR-R, JP-E, and LS-S. Wrote the paper: JD-F and PP-S. Revised the manuscript: JD-F and PP-S. Contributed reagents/materials/analysis tools: NM-G and LS-S. All authors contributed to the article and approved the submitted version.
