# Supplementary material for: Identification of genetic and biochemical mechanisms associated with heat shock and heat stress adaptation in grain amaranths
Source: Front Plant Sci. 2023 Feb 2;14:1101375. doi: 10.3389/fpls.2023.1101375 (PMC9932720; doi:10.3389/fpls.2023.1101375)
Supplement: Supplementary file 1 [file Table_1.docx]

**Table S1. Oligonucleotides utilized for the qPCR assays used quantify the expression of unknown function genes in heat-shocked grain amaranth^a^ plants.**

| **Experimental objective** | **Oligonucleotide sequence** | **Oligonucletide**  **size (bp)b** |
| --- | --- | --- |
| **Quantitative gene expression (qPCR) in grain amaranths^b^** |  |  |
| *Ah2880* (F) | TTAAACCTTCAACGCCAACTGATCTT | 26 |
| *Ah2880* (R) | TTAAACCTTCAACGCCAACTGATCTT | 26 |
| *AhHAB4-PAI-1* (F) | CTACACCTCTTCCACCTTCG | 20 |
| *AhHAB4-PAI-1* (R) | TCAAGAGCTCAAAGTTACTGC | 21 |
| *AhRIP* (F) | CTGGACGAGATATGCCGAA | 19 |
| *AhRIP* (R) | CAGCAGATAGGTTCCACGACT | 21 |
| *AhBAMY* (F) | GAGCTTACAAGTCACAATACCAC | 23 |
| *AhBAMY* (R) | GCAAGCTCAAAAGGTTTATG | 20 |
| *AhDGR2* (F) | AAGGCCTACGAACACTAACG | 20 |
| *AhDGR2* (R) | GGAAAAGGTGGATACAAACG | 20 |
| *AhTIL* (F) | TGGTAGTAGGTAGTCGGCAG | 20 |
| *AhTIL* (R) | AACAAACTACACAAGACTCCTCA | 23 |
| *AhERD* (F) | GCAACACATGAAGGGATGG | 19 |
| *AhERD* (R) | TGGGACTTGTATTCAGCAGC | 20 |
| *AhOEE* (F) | GTGCCGGCTTAGACTCTCC | 19 |
| *AhOEE* (R) | GCTTGCTTAATTGTATAATCGTCC | 24 |
| *AhActinACT7* (F) | CGTGACCTGACTGATTACCTTA | 22 |
| *AhActinACT7* (R) | GCTCGTAGTTCTTCTCAATGGC | 22 |
| *AhβTubulin* (F) | TCTCAGCAGTATGTCTCCCTCA | 22 |
| *AhβTubulin* (R) | TCTACTTCTTTGGTGCTCATCTT | 23 |

^a^Grain amaranths *= Amaranthus hypochondriacus, A. cruentus* and *A. caudatus.*

^b^bp = base pairs.
