# Supplementary material for: Identification of genetic and biochemical mechanisms associated with heat shock and heat stress adaptation in grain amaranths
Source: Front Plant Sci. 2023 Feb 2;14:1101375. doi: 10.3389/fpls.2023.1101375 (PMC9932720; doi:10.3389/fpls.2023.1101375)
Supplement: Supplementary file 2 [file Table_2.docx]

**Table S2.** Data obtained from the HPLC analysis optimized for phenylpropanoids and phenolic acid compounds of leaf extracts obtained from *Amaranthus hypochondriacus* plants subjected to heat shock (HS) treatment and recovery.

| **RT^1,2^** | | | **AREA^3^** | | |
| --- | --- | --- | --- | --- | --- |
| **Control**  **(0 h)** | **Heat shock**  **(30 h)** | **Recovery**  **(3 days)** | **Control**  **(0 h)** | **Heat shock**  **(30 h)** | **Recovery**  **(3 days)** |
| 2.907 | 3.032 |  | 22.26858 | 25.34481 |  |
| 3.748 | 3.833 | 3.863 | **120.72457** | 13.89572 | 59.14769 |
|  | 4.008 |  |  | 24.04649 |  |
|  | 4.536 |  |  | **51.77081** |  |
|  | 4.999 | 4.929 |  | 21.86486 | **91.51053** |
|  | 6.600 |  |  | 21.42739 |  |
|  | 7.342 | 7.269 |  | 10.18424 | **109.56554** |
|  | 7.595 | 7.817 |  | 19.76770 | 21.00314 |
|  | 9.454 | 9.846 |  | 13.29234 | **41.71541** |
|  | 12.885 | 12.639 |  | 10.45335 | 34.37380 |
| 14.730 |  | 14.835 | 35.95689 |  | 10.55137 |
|  | 15.315 | 15.257 |  | **298.11633** | 21.67250 |
| 16.530 |  |  | 14.94151 |  |  |
|  |  | 18.812 |  |  | **50.83702** |
| 26.130 | 26.776 | 25.985 | 17.26307 | **51.77396** | 15.64095 |
|  |  | 27.041 |  |  | 36.85867 |
| 34.586 |  |  | **86.10921** |  |  |
|  |  |  |  |  |  |
|  | 35.841 | 36.273 |  | **249.62581** | 27.91662 |
|  | 50.916 | 50.210 |  | 16.96386 | 18.40286 |
|  | 51.272 | 50.970 |  | 28.94011 | 13.46727 |
|  | 52.529 |  |  | 12.92969 |  |
|  | 53.147 | 53.166 |  | **56.22745** | 14.84076 |
|  | 54.170 |  |  | **46.86243** |  |
|  | 54.723 | 55.061 |  | 36.93850 | 10.19368 |
|  | 59.856 |  |  | 24.13736 |  |
| 76.585 |  |  | 10.82940 |  |  |
| 80.104 | 80.106 | 80.171 | 16.79063 | 15.39761 | 14.19273 |
| 83.327 | 83.333 | 83.413 | 54.37728 | 23.67194 | 20.31238 |
| 84.981 | 84.995 | 85.043 | 28.99882 | 22.55250 | 14.58208 |
| 85.979 |  |  | **90.01318** |  |  |
| 86.768 | 86.789 |  | **277.99399** | **258.49542** |  |
| 87.404 |  |  | 22.40209 |  |  |
| 88.180 | 88.395 |  | **63.67887** | 52.83945 |  |
| 88.756 | 88.833 |  | **108.67586** | 19.66799 |  |
| 89.287 | 89.161 |  | 37.53354 | 42.89376 |  |

^1^The detection was performed at 280 nm.

^2^RT = retention time

^3^Area= peak area
