# Supplementary material for: Identification of genetic and biochemical mechanisms associated with heat shock and heat stress adaptation in grain amaranths
Source: Front Plant Sci. 2023 Feb 2;14:1101375. doi: 10.3389/fpls.2023.1101375 (PMC9932720; doi:10.3389/fpls.2023.1101375)
Supplement: Supplementary file 3 [file Table_3.docx]

**Table S3.** Data obtained from the HPLC analysis optimized for flavonoid compounds of leaf extracts obtained from *Amaranthus hypochondriacus* plants subjected to heat shock (HS) treatment and recovery.

**Treatment:** Control (0 h HS)

| **280 nm^1^** | | **320 nm** | | **360 nm** | |
| --- | --- | --- | --- | --- | --- |
| **RT^2^** | **AREA^3^** | **RT** | **AREA** | **RT** | **AREA** |
| 1.376 | **531.41650** | 1.303 | 81.77969 | 1.495 | 313.97897 |
|  |  |  |  | 2.458 | 51.00079 |
|  |  |  |  | 4.622 | 349.88892 |
| 1.491 | 613.41998 | 1.495 | **1052.37720** |  |  |
|  |  | 2.247 | 49.03429 |  |  |
| 2.458 | 72.98238 | 2.458 | 156.92618 |  |  |
|  |  | 3.519 | 83.78336 | 5.261 | 1459.27991 |
| 4.644 | 410.68997 | 4.629 | **965.86676** |  |  |
| 5.261 | 1830.33447 | 5.261 | **4046.52197** |  |  |
| 6.746 | 611.49664 | 6.747 | **1359.01965** | 6.745 | 76.37207 |
| 7.279 | 418.57150 | 7.279 | **1069.69324** | 7.279 | 321.63580 |
|  |  | 8.833 | 41.44429 |  |  |
| 9.099 | 60.57811 |  |  |  |  |
| 10.530 | 66.59798 | 10.532 | 82.67732 |  |  |
| 11.182 | 63.76609 | 11.182 | 103.16221 | 11.182 | 46.69454 |
| 12.665 | 42.98597 |  |  |  |  |
|  |  | 31.533 | 54.73815 | 31.532 | 64.33018 |
| 32.189 | 63.20460 | 32.190 | 75.41282 | 32.190 | 89.77484 |
| 32.713 | 1027.08105 | 32.712 | 1352.07605 | 32.704 | **1643.46057** |
|  |  | 33.561 | 41.28643 |  |  |
| 34.063 | 81.34032 | 34.063 | 136.78720 | 34.064 | 313.40121 |
|  |  | 34.447 | 51.85338 | 34.445 | 116.62234 |
| 35.302 | 48.32903 | 35.302 | 65.57994 | 35.302 | 151.02475 |
|  |  |  |  | 35.561 | 65.83430 |
| 36.158 | 1995.42041 | 36.158 | 3176.18701 | 36.158 | **5988.11035** |
| 37.564 | 462.03958 | 37.561 | 705.70160 | 37.558 | **1272.91248** |
| 38.668 | 76.16045 | 38.670 | 56.82478 | 38.669 | 228.66969 |
| 39.049 | 54.40817 | 39.045 | 60.16812 | 39.041 | 76.83513 |
|  |  |  |  |  |  |

**Treatment:** Heat shock, 30 h

| **280 nm** | | **320 nm** | | **360 nm** | |
| --- | --- | --- | --- | --- | --- |
| **RT** | **AREA** | **RT** | **AREA** | **RT** | **AREA** |
|  |  | 1.303 | 121.25359 | 1.299 | 50.76139 |
| 1.373 | **815.76050** |  |  |  |  |
| 1.491 | 1001.69623 | 1.495 | **1672.77258** | 1.495 | 519.84082 |
| 2.006 | 42.76606 | 2.013 | 46.93221 |  |  |
| 2.247 | 41.30679 | 2.248 | 97.56607 |  |  |
| 2.461 | 46.62793 | 2.460 | 94.22965 |  |  |
|  |  | 2.922 | 67.20267 |  |  |
| 3.730 | **938.00696** |  |  |  |  |
| 4.710 | 221.33801 | 4.704 | 431.09491 | 4.704 | 155.63341 |
| 5.255 | 4434.73291 | 5.255 | **9893.56445** | 5.256 | 3530.19482 |
|  |  | 5.971 | 65.42302 |  |  |
| 6.745 | 1953.14099 | 6.746 | **4433.50439** | 6.745 | 198.38611 |
|  |  |  |  | 6.936 | 81.14489 |
| 7.275 | 1030.44580 | 7.276 | **2711.11279** | 7.276 | 769.35242 |
| 7.938 | 42.89949 |  |  |  |  |
| 8.825 | 168.64053 | 8.828 | 229.42734 | 8.823 | 156.75168 |
| 9.077 | 255.36134 | 9.089 | 330.26712 |  |  |
| 10.038 | 130.81189 | 10.029 | 218.78217 |  |  |
| 10.503 | 299.23877 | 10.503 | 470.83609 |  |  |
| 11.174 | 496.47595 | 11.174 | **778.60364** | 11.174 | 378.19983 |
| 11.336 | 66.22070 | 11.334 | 80.54883 |  |  |
| 12.600 | 88.90420 |  |  |  |  |
|  |  | 13.278 | 40.19399 |  |  |
| 14.148 | 44.60012 | 14.149 | 67.17859 | 14.147 | 49.95953 |
|  |  | 14.840 | 48.83849 | 18.611 | 85.17443 |
| 16.361 | 157.89374 | 16.361 | 259.53964 |  |  |
| 18.611 | 178.93794 | 18.611 | 275.63535 |  |  |
| 27.935 | 64.79160 | 27.935 | 109.95738 | 27.935 | 102.32819 |
| 31.547 | 77.11829 | 31.540 | 54.60506 | 31.539 | 63.47657 |
| 32.196 | 79.53855 | 32.196 | 98.54230 | 32.197 | 118.09079 |
| 32.718 | 2027.53235 | 32.717 | 2613.56787 | 32.714 | **2891.65796** |
|  |  |  |  | 33.288 | 49.14170 |
| 33.567 | 85.42568 | 33.567 | 103.14066 | 33.566 | 92.86297 |
|  |  |  |  | 34.076 | 50.82318 |
|  |  | 34.460 | 44.48515 | 34.459 | 100.35562 |
| 35.319 | 64.58566 | 35.319 | 74.65387 | 35.319 | 169.46790 |
| 36.190 | 4014.17798 | 36.190 | 6346.77881 | 36.190 | **11728.8** |
|  |  |  |  | 37.617 | **1350.00195** |
| 37.637 | 507.70261 | 37.628 | 766.95483 |  |  |
|  |  |  |  | 38.714 | 106.54417 |
| 39.086 | 59.61449 | 39.084 | 104.41913 | 39.081 | 131.09152 |
|  |  |  |  |  |  |

**Treatment:** Recovery, 3 days

| **280 nm** | | **320 nm** | | **360 nm** | |
| --- | --- | --- | --- | --- | --- |
| **RT** | **AREA** | **RT** | **AREA** | **RT** | **AREA** |
| 1.374 | 91.15411 | 1.302 | 50.94643 |  |  |
| 1.488 | 1757.95398 | 1.492 | **2806.81152** | 1.492 | 849.42755 |
| 1.950 | 168.47942 | 1.950 | 321.18881 | 1.950 | 93.07581 |
|  |  | 2.250 | 50.97878 | 2.686 | 286.41739 |
| 2.686 | 185.57541 | 2.686 | **419.50867** |  |  |
| 3.864 | 199.20413 | 3.866 | 259.49036 |  |  |
|  |  | 4.596 | **1043.56042** | 4.581 | 360.22308 |
| 4.692 | 407.79312 |  |  |  |  |
| 5.249 | 2073.01172 | 5.249 | **4693.89600** | 5.249 | 1662.42603 |
| 5.956 | 62.73143 | 5.953 | 97.27711 |  |  |
| 6.734 | 143.36227 | 6.735 | 330.93695 |  |  |
| 6.929 | 140.36888 | 6.929 | 148.23936 | 6.929 | 279.31433 |
| 7.270 | 64.84976 | 7.270 | 215.98384 | 7.271 | 58.13387 |
| 7.913 | 65.65686 | 7.905 | 40.19398 | 7.905 | 42.79187 |
| 8.931 | 48.30915 | 8.940 | 76.25490 |  |  |
|  |  | 10.513 | 52.29953 |  |  |
| 11.178 | 58.66282 | 11.177 | 93.92051 | 11.178 | 41.25450 |
|  |  | 11.919 | 55.98700 |  |  |
|  |  | 12.773 | 45.34400 |  |  |
| 32.714 | 112.44850 | 32.712 | 154.36903 | 32.696 | 196.49620 |
| 36.148 | 275.90759 | 36.148 | 437.30585 | 36.148 | **829.64410** |
|  |  |  |  | 37.578 | 143.31026 |
| 37.674 | 67.37195 | 37.666 | 94.18516 |  |  |
|  |  |  |  |  |  |

^1^The detection was performed using 3 different wavelengths: 280, 320 and 360 nm.

^2^RT = retention time

^3^Area= peak area
