# Supplementary figures and images for: Identification of genetic and biochemical mechanisms associated with heat shock and heat stress adaptation in grain amaranths
Source: Front Plant Sci. 2023 Feb 2;14:1101375. doi: 10.3389/fpls.2023.1101375 (PMC9932720; doi:10.3389/fpls.2023.1101375)

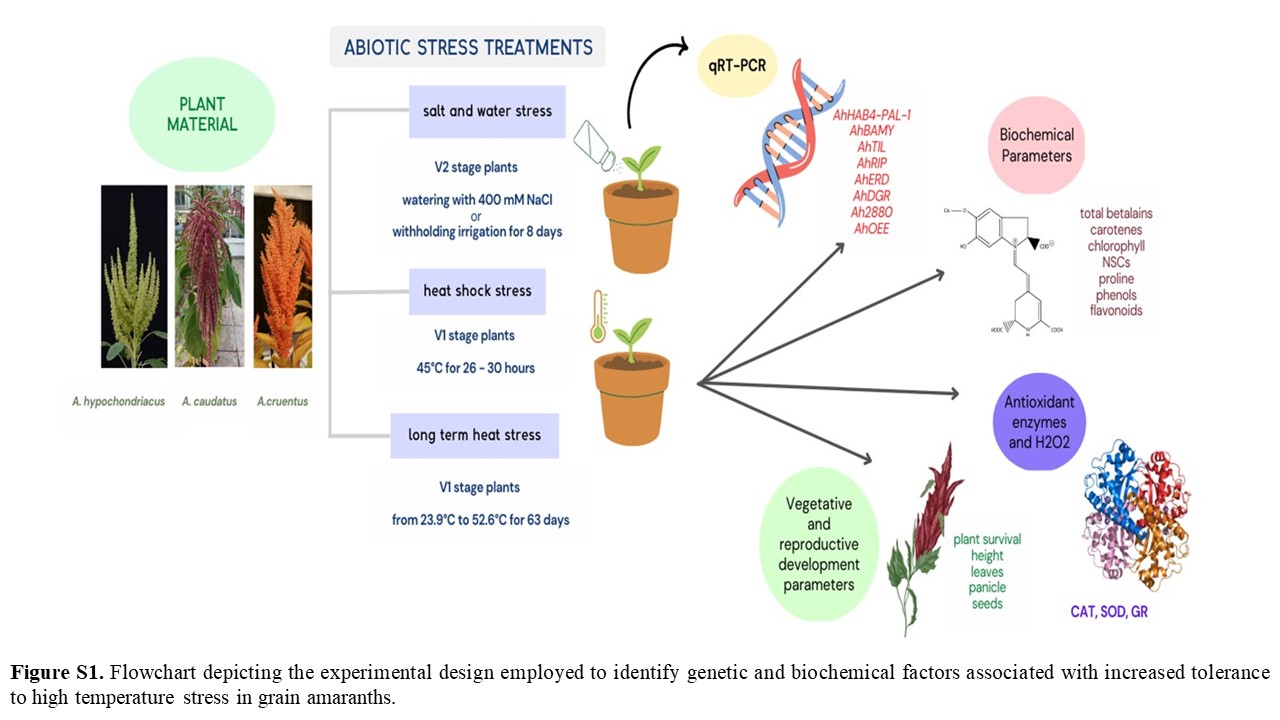

Supplement: Supplementary file 4 [file Image_1.jpeg]
